# A post-hoc analysis of risk factors for poor quality of life after surgical treatment of spondylodiscitis

**DOI:** 10.1038/s41598-024-79828-8

**Published:** 2024-11-17

**Authors:** Krishnan Sircar, Dorothee Jochimsen, Charlotte Meyer-Schwickerath, Norma Jung, Nikolaus Kernich, Peer Eysel, Ayla Yagdiran

**Affiliations:** 1 Department of Orthopaedics and Trauma Surgery, University Hospital Cologne, Faculty of Medicine, University of Cologne, Kerpener Strasse 62, 50937 Cologne, Germany; 2 Department I of Internal Medicine, University Hospital Cologne, Faculty of Medicine, University of Cologne, Kerpener Strasse 62, 50937 Cologne, Germany

**Keywords:** Proms, Spondylodiscitis, Risk factors, Quality of life, Malignancy, Infectious diseases, Prognosis, Quality of life, Outcomes research, Risk factors

## Abstract

Patient-reported outcome measures for quality of life (QoL) like the Oswestry-Disability-Index (ODI) are useful for a quick, comprehensive and comparable evaluation of spondylodiscitis treatment. While it is known that even after successful surgical treatment, the QoL of spondylodiscitis patients remains below that of the general population, risk factors for a poor outcome have not been described so far. We did a retrospective post-hoc analysis of 130 surgically treated spondylodiscitis patients from 2008 to 2022. Biographical, clinical und QoL data were prospectively collected before (T_0_) and one year (T_1_) after surgery. The primary outcome was QoL at T_1_. Patients were grouped according to their ODI score at T_1_ (Group 1: ODI < 35, favorable QoL; Group 2: ODI ≥ 35, poor QoL) and analyzed for risk factors present at T_0_. Poor QoL at T_1_ occurred in 51 patients (39%). Multivariate analysis identified a higher preoperative leg pain (odds ratio: 1.2) and the presence of a malignant disease (odds ratio: 1.9) as independent preoperative risk factors for a poor QoL at T_1_. Spondylodiscitis patients with malignancy and/or preoperative leg pain may be at increased risk for poor QoL after surgical treatment. These results can be used to individualize patient information and provide a better assessment of prognosis before surgery.

**Supplementary Information:**

The online version contains supplementary material available at 10.1038/s41598-024-79828-8.

## Introduction

Evaluating the treatment of spondylodiscitis (SD) with the help of patient-reported outcome measures such as Quality of Life (QoL) has gained recognition over the past decade^1–6^. They allow for a quick, comprehensive and comparable evaluation of outcome. A number of questionnaires have been developed to measure QoL, such as the Core Outcome Measures Index (COMI), the Short-Form 36 (SF-36) and the European Quality of Life Questionnaire (EQ-5D). The Oswestry Disability Index (ODI) was proposed by Fairbank et al. in 1980^7^. It focuses on the functional status of patients and the disability to perform activities of daily life. It has become a gold standard for evaluating a variety of spinal procedures and conditions, including SD^8^. While the QoL of SD patients improves after healing, it remains below that of the general population and is comparable to the QoL of chronic low back pain patients^1,3^. When informing SD patients on their prognosis, knowledge of risk factors for an unfavorable outcome is essential. However, risk factor analyses for an adverse course of treatment often focus on outcomes such as mortality, relapse or neurologic deficits^4,9^. Relying on these rather extreme factors alone without consideration of QoL may lead to an underestimation of unfavorable outcome. Recently, ODI threshold values for a favorable and unfavorable long-term outcome of SD have been published^10^, but risk factors that predispose patients to a high ODI are unknown so far. The aim of this study is to identify risk factors for an unfavorable outcome in terms of ODI to improve the assessment of the prognosis of spondylodiscitis.

## Materials and methods

### Ethics Statement

Approval by the Ethics Committee of the University Hospital of Cologne (No. 09-182) was obtained prior to inclusion. The study was conducted in accordance with the Declaration of Helsinki.

### Patient selection

From January 2008 until December 2022, all adult patients with SD were prospectively enrolled in an SD registry at a tertiary referral center. Written consent from each patient was obtained. Inclusion criteria were suspected SD in patients with characteristic clinical features such as back and/or leg pain and typical MRI/CT findings such as contrast enhancement of discs and/or vertebral bodies, abscesses, and vertebral body destruction. All cases were reviewed by a panel including an orthopedic surgeon and an infectious diseases specialist. Patients who were assigned to conservative treatment were excluded. Only patients with a follow-up of at least one year and complete QoL data upon inclusion and at follow-up were included in this study. Another exclusion criterion was the need for emergency surgery because of severe sepsis and/or acute neurologic deficits. These patients were not considered able to reliably complete a QoL questionnaire before surgery. Patients that were unable to complete a QoL questionnaire because of dementia were excluded as well.

### Surgical treatment

Indications for surgical treatment included spinal instability, manifest or impending neurologic deficits, failure of conservative treatment and immobilizing pain despite analgetic therapy. Surgery was carried out as dorsal instrumentation, combined with debridement and dorsal interbody fusion if possible (PLIF or TLIF technique). When dorsal interbody fusion was not possible because of the extent of bone destruction, a two-stage approach with secondary anterior interbody fusion or vertebral body replacement with cages or iliac crest grafts was chosen.

### Data collection

QoL data were prospectively collected. To assess QoL, the Oswestry Disability Index (ODI) was surveyed from each patient. The ODI consists of 10 items that result in a score from 0 to 100 (supplemental Table 1). A lower score demonstrates a higher QoL. Data were collected preoperatively (T_0_) and one year after surgery (T_1_). In addition, the following variables were recorded for all patients: age, sex, duration of hospital stay, microbiological pathogens, bloodstream infection, affected spine segment, preoperative neurological deficits, post-operative recurrence, comorbidities (measured with Charlson Comorbidity Index^11^), concomitant endocarditis, preoperative back pain measured with visual analogue scale (VAS) from 0 to 10, preoperative leg pain, previous spine surgery or spine injections and the American Society of Anesthesiologists (ASA) score.

Based upon their ODI score at T1, patients were either assigned to Group 1 (ODI < 35, favorable QoL) or Group 2 (ODI ≥ 35, unfavorable QoL).

Additionally, a sub-group analysis of the ODI of patients with complete follow-up data after two years (T_2_) was carried out, to analyze changes of ODI over a longer period of time.

### Statistical analysis

Patient demographics and clinical factors were analyzed descriptively and are reported as either mean (± standard deviation), median (interquartile range) or number (percentage). The distribution of independent variables between Groups 1 and 2 was analyzed by univariate tests: Student’s t-Test for continuous variables, Mann-Whitney-U-Test for ordinal variables, and Chi-Square-Test for dichotomous variables. Level of significance was set at *p* ≤ 0.05. All variables with a p-value ≤ 0.1 in univariate testing were included in a binary logistic regression model with stepwise backward elimination. Odds ratios for variables that were significant in logistic regression (*p* ≤ 0.05) are reported with 95% confidence intervals. IBM SPSS Statistics 22 was used for the statistical analysis.

## Results

A total of 130 patients were included in the study, with 84 (65%) male patients and 46 (35%) female patients. The mean age of the study population was 65 years (SD: ±11.6; 95%-confidence-interval: 63–67), ranging from 20 years to 84 years. In 57 patients (44%), *S. aureus* was identified as the pathogen causing the infection. The mean ODI of the whole cohort was 74 at T_0_ and 29 at T_1_.

Seventy-nine of the 130 patients (61%) had an ODI score of < 35 (mean: 13) at T_1_ and were assigned to Group 1, indicating a favorable outcome. The remaining 51 patients (39%) had a ODI score of ≥ 35 (mean: 54) at T_1_ and were assigned to Group 2. Table 1 shows the results of the descriptive analysis for both groups and the results of the univariate analysis of potential risk factors. Mean ODI score at T_0_ showed no significant difference between both groups (74 vs. 75; *p* = 0.92). Univariate analysis identified the presence of endocarditis, a higher preoperative leg pain and a higher ASA Score at T_0_ as significant risk factors for an unfavorable ODI score at T_1_.

**Table 1:** Patient demographics and clinical factors at T_0_ for Group 1 (patients with ODI < 35) and Group 2 (patients with ODI ≥ 35), as well as p-values of univariate testing. Data is reported as either mean (± standard deviation), median (interquartile range) or absolute number (percentage). *P ≤ 0.05; **P ≤ 0.1.

Factor	Group 1 (ODI < 35) *n* = 79	Group 2 (ODI ≥ 35) *n* = 51	*P*-Value
Male sex, n (%)	54 (68)	30 (59)	0.27
Bacteraemia, n (%)	18 (23)	17 (33)	0.19
Staph. aureus, n (%)	35 (44)	22 (42)	0.85
Diabetes, n (%)	10 (13)	10 (20)	0.28
Immunosuppression, n (%)	5 (6)	4 (8)	0.74
Malignancy, n (%)	10 (13)	12 (24)	0.10**
COPD^a^, n (%)	7 (9)	3 (6)	0.53
Heart failure, n (%)	9 (11)	5 (10)	0.78
Chronic renal failure, n (%)	9 (11)	6 (12)	0.95
Endocarditis, n (%)	0 (0)	3 (6)	0.03*
Alcohol abuse, n (%)	5 (6)	3 (6)	0.92
Drug abuse, n (%)	0 (0)	4 (8)	0.12
Osteoporosis, n (%)	4 (5)	0 (0)	0.12
Neurologic impairment, n (%)	13 (16)	12 (24)	0.32
Psoas abscess, n (%)	16 (20)	11 (22)	0.86
Spinal abscess, n (%)	40 (51)	23 (45)	0.54
No pathogen detected, n (%)	24 (30)	11 (22)	0.27
Age (a)	65 (12.3)	65 (10.5)	0.91
ODI at T_0_	74 (20.8)	75 (16.9)	0.92
Time to surgery (d)	6.2 (7.1)	7.6 (7.3)	0.29
Length of stay (d)	31.3 (16.7)	34.3 (19.9)	0.36
BMI^b^ (kg/m²)	25.8 (5.3)	26.5 (6.2)	0.51
CRP^c^ (mg/L)	96 (105)	80 (92)	0.40
WBC^d^ (/µL)	10 (4.6)	9.2 (3.4)	0.27
Creatinine (mg/dL)	1.1 (0.7)	0.9 (0.5)	0.99
Hb^e^ (g/dL)	11.9 (1.9)	11.5 (1.8)	0.24
Serum albumin (g/dL)	34.7 (7.8)	35.8 (7.2)	0.41
Number of comorbidities	0.9 (1.1)	1 (1)	0.57
Affected segments	1.3 (0.5)	1.3 (0.7)	0.92
Instrumented segments	1.9 (1.4)	2.1 (1.5)	0.43
Back pain (VAS^f^)	8.5 (7–10)	8 (7–10)	0.15
Leg pain (VAS)	4 (0–7)	7.5 (3.8-9-3)	0.001*
ASA score^g^	2 (2–3)	3 (2–3)	0.01*
CCI^h^	3 (2–4)	3 (2–4)	0.79

a: Chronic Obstructive Lung Disease; b: Body Mass Index; c: C-reactive Protein; d: White Blood Cell Count; e: Hemoglobin; f: Visual Analogue Scale; g: American Society of Anesthesiologists; h: Charlson Comorbidity Index.

**Fig. 1:**
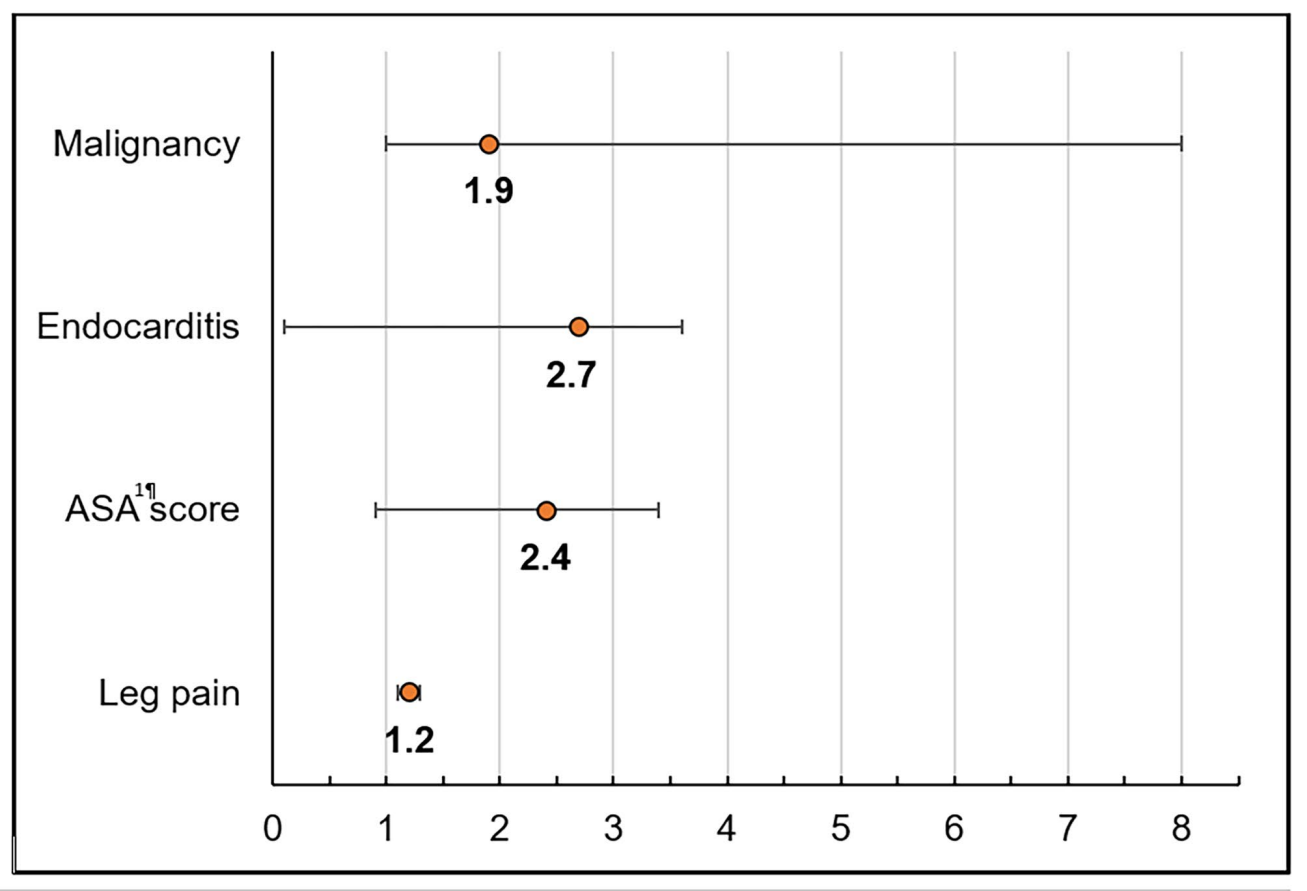
Forest Plot of the odd ratios of all factors of the multivariable analysis.

In the multivariate analysis, preoperative leg pain and the presence of a malignant disease were significant risk factors for an unfavorable ODI score at T_1_ (Fig. 1; Table 2).

**Table 2:** Results of the multivariable analysis. *P ≤ 0.05.

Factor	*P*-Value	Odds Ratio [95%-CI]
Leg pain	0.004*	1.2 [1.1–1.3]
ASA^a^ score	0.1	2.4 [0.9–3.4]
Endocarditis	0.9	2.7 [0–3.6]
Malignancy	0.04*	1.9 [1–8]

^a^: American Society of Anesthesiologists.

Patients with (*n* = 22) and without (*n* = 108) a malignant disease showed no significant difference in their mean ODI score at T_0_ (68 vs. 76, *p* = 0.08). Supplemental Table 2 details neoplasm entities (solid vs. hematologic) and the status of disease activity of the 22 patients with a malignant disease.

Follow-up data of 82 (63%) patients were available at T_2_, with 54 patients in Group 1 and 28 patients in Group 2. The mean ODI at T_2_ of Group 1 was 16, the mean ODI of Group 2 was 53. The differences to the mean ODI at T_1_ were not significant for both groups (*p* = 0.16 for Group 1 and *p* = 0.62 for Group 2).

## Discussion

In this post-hoc analysis of data from 130 patients of a prospective SD registry, we aimed to identify risk factors that predispose patients to an unfavorable outcome in terms of ODI. We found that preoperative leg pain and the presence of a malignant disease were associated with an ODI ≥ 35, indicating an unfavorable outcome one year after treatment.

With a predominance of male sex, a mean age of 65 years and *Staph. aureus* as the most prevalent pathogen, our cohort is typical for SD^12,13^. Our cohort showed a significant improvement in terms of ODI after treatment, which is in line with other studies^2,5^. However, approximately one third of our study population had an unfavorable outcome in terms of ODI. This confirms findings by Stoop et al.^6^, who found that 44% of their patients had an ODI of > 20 five years after diagnosis, and underlines the great impact SD has on patients’ lives.

A number of studies were able to identify risk factors that influence the outcome after treatment of SD. Aagaard et al.^14^ found that substance abuse was associated with an increased mortality one year after diagnosis. Ukon et al.^15^ identified chronic pulmonary disease, diabetes mellitus, gram-negative bacteria, pyogenic osteoarthritis, high preoperative white blood cell count, and low preoperative platelet count as risk factors for severe postoperative complications - such as intensive care unit treatment, need for revision or death - after surgical treatment of SD. The presence of *Staph. aureus* and a prolonged duration of symptoms before treatment were risk factors for treatment failure - defined as death, relapse, neurologic sequelae or need for revision surgery – in a study by Gupta et al.^9^. However, in patients with apparently successful SD treatment who do not suffer from these well-defined endpoints, assessment of outcome can be difficult^3^. Measuring QoL with standardized questionnaires may serve as an objective, comparable and efficient tool to comprehensively measure outcome. To the best of our knowledge, this is the first study that analyses risk factors for a low QoL after surgical treatment of SD. We chose the ODI to evaluate QoL because it is the only spine-specific questionnaire and superior in the ability to diagnose clinical improvement^16^. The ODI was developed by Fairbank et al. in 1980^7^ and was originally intended to be disease-specific for chronic low back pain. It has since been used to measure QoL in a variety of spinal diseases, such as degenerative disc disease, disc herniation, spinal stenosis and SD^6,17^. The ODI offers good reliability, validity and responsiveness to change^3^. However, in order to be able to properly contextualize the ODI score, threshold values defining satisfactory and unsatisfactory outcome are mandatory. For example, in patients with chronic low back pain, a cut-off ODI value of *12* separates patients with disability^18^ from patients without disability, whereas a cut-off value of *30* indicates whether patients receiving inpatient care for low back pain are dischargeable or not. In patients with adult spinal deformity, a cut-off value of *18* distinguishes between acceptable and unacceptable QoL^19^. Yagdiran et al.^10^ reported a cut-off value of *35* as a threshold for favorable and unfavorable outcome after surgical treatment of SD. We used this cut-off value to determine risk factors for an unfavorable outcome.

In our multivariable analysis, the presence of malignancy was found to be an independent risk factor for an unfavorable outcome. While it is known that malignant diseases are associated with a higher risk for SD^20^, data on their influence on the long-term course of the disease is rare. Akiyama et al.^21^ found an increased in-hospital mortality in patients with SD and malignancy. Conversely, in a retrospective, Scottish study, Veljanoski et al.^22^ found a better outcome (graded as recovery, residual symptoms or neurological deficits, paraplegia or death) in patients with a history of malignancy. The authors attributed this paradox finding to the fact that cancer patients in Scotland can be quickly triaged and referred to a hospital via a hotline if they feel unwell. It is important to note that in our cohort, malignancy was not per se associated with poor *initial* QoL. However, the presence of a malignant tumor and its therapy in form of chemotherapy or radiation is associated with immunosuppression. This in turn impacts the ability to heal an infection. Also, the addition of a second severe disease (spondylodiscitis) to a malignoma may overcompensate coping resources and therefore lead to a lower QoL. We found no influence of neoplasm entity or disease activity on QoL, but because of the overall low number of patients in the subgroups (s. Supplemental Table 2), no conclusion should be drawn from the lack of statistical significance. Surprisingly, preoperative leg pain but not back pain was another independent risk factor for a low ODI one year after surgery in this study. Whereas back pain may be more easily addressed by spinal stabilization, leg pain can be a sign of radicular affection, which may hint to a higher degree of osseous destruction or even epidural abscess^23^. Despite decompression and stabilization, leg pain may be persistent after surgery, because nerve tissue has a lower potential for rehabilitation. In addition, the formation of peridural scar tissue after decompression could contribute to persistent postoperative leg pain^24^. The significance of leg pain in the evaluation of SD has been previously described. Sircar et al.^25^ found a (weak) correlation between preoperative leg pain and the severity of SD. Yagdiran et al.^26^ found an association with lower pretreatment leg pain and favorable outcome of SD. Leg pain is easily assessable and quantifiable and may therefore be an effective and simple item for estimating posttreatment outcome of SD. As has been shown previously^1^, QoL of SD patients seems to reach a plateau after one year and remains stable after two years. Our study further demonstrates that this is true for both unfavorable and favorable QoL.

In this study, age and multimorbidity were not significant risk factors for unfavorable QoL after SD. SD mostly affects elderly people^27^, and consequently, our study population predominantly consisted of elderly people with a quite narrow age spectrum. The effect of younger age on QoL should therefore be further investigated in a more age-diverse study population. Accordingly, multimorbidity, as measured by the Charlson Comorbidity Index (CCI), was rather high in our study population, with only 22 patients (17%) percent having an CCI of < 2. Therefore, conclusions regarding the impact of multimorbidity on QoL after SD should be drawn with caution.

### Limitations

This study has several limitations. Our cohort is from a tertiary referral center specialized in spine surgery. The population may therefore consist of especially severe cases and may not be representative of general SD patients.

In addition, the number of patients being transferred from other hospitals to our institution for further treatment was quite high and most of culture negative SD cases had received antibiotic treatment prior to sampling. We found no difference in culture positive versus culture negative cases in regards to QoL, but this relevant bias must be taken into consideration when interpreting the effect of pathogen detection on QoL in our study population.

The exclusion of patients who needed emergency surgery, had severe sepsis before treatment or suffered from dementia may introduce a selection bias as these patients are likely to have a lower QoL before and after treatment.

## Conclusions

Spondylodiscitis patients with an underlying malignant disease and/or preoperative leg pain may be at increased risk for poor quality of life after surgical treatment. The results of this study can be used to individualize patient information and provide a better assessment of prognosis before surgery.

## Electronic supplementary material

Below is the link to the electronic supplementary material.

Supplementary Material 1

Supplementary Material 2

## Data Availability

The datasets used and analyzed during the current study are available from the corresponding author on reasonable request.
