# Publisher Correction: A post-hoc analysis of risk factors for poor quality of life after surgical treatment of spondylodiscitis

**DOI:** 10.1038/s41598-025-86029-4

**Published:** 2025-02-05

**Authors:** Krishnan Sircar, Dorothee Jochimsen, Charlotte Meyer-Schwickerath, Norma Jung, Nikolaus Kernich, Peer Eysel, Ayla Yagdiran

**Affiliations:** 1 Department of Orthopaedics and Trauma Surgery, University Hospital Cologne, Faculty of Medicine, University of Cologne, Kerpener Strasse 62, 50937 Cologne, Germany; 2 Department I of Internal Medicine, University Hospital Cologne, Faculty of Medicine, University of Cologne, Kerpener Strasse 62, 50937 Cologne, Germany

Correction to: *Scientific Reports* 10.1038/s41598-024-79828-8, published online 17 November 2024

The original version of this Article contained errors.

References 14 – 25 were omitted and are listed below.

14. Aagaard, T., Roed, C., Dahl, B. & Obel, N. Long-term prognosis and causes of death after spondylodiscitis: A Danish nationwide cohort study. *Infect. Dis.* **48**, 201–208 10.3109/23744235.2015.1103897 (2016).
15. Ukon, Y. et al. Preoperative risk factors affecting outcome in surgically treated pyogenic spondylodiscitis. *Glob. Spine J.* **13** 10.1177/21925682221077918 (2023).
16. Johnsen, L. G. et al. Comparison of the SF6D, the EQ5D, and the oswestry disability index in patients with chronic low back pain and degenerative disc disease. *BMC Musculoskelet. Disord.* **14**, 1–9 10.1186/1471-2474-14-148/FIGURES/4 (2013).
17. Power, J. D. et al. Determining minimal clinically important difference estimates following surgery for degenerative conditions of the lumbar spine: analysis of the Canadian Spine Outcomes and Research Network (CSORN) registry. *Spine J.* **23**, 1323–1333 10.1016/J.SPINEE.2023.05.001 (2023).
18. Tonosu, J. et al. The normative score and the cut-off value of the Oswestry Disability Index (ODI). *Eur. Spine J.* **21**, 1596–1602 10.1007/S00586-012-2173-7/FIGURES/4 (2012).
19. Mannion, A. F. et al. What level of symptoms are patients with adult spinal deformity prepared to live with? A cross-sectional analysis of the 12-month follow-up data from 1043 patients. *Eur. Spine J.* **29**, 1340–1352 10.1007/S00586-020-06365-Z/TABLES/6 (2020).
20. Giordan, E., Marton, E., Scotton, G. & Canova, G. Outcomes and risk factors for spontaneous spondylodiscitis: Case series and meta-analysis of the literature. *J. Clin. Neurosci.* **68**, 179–187 10.1016/J.JOCN.2019.06.040 (2019)
21. Akiyama, T. et al. Incidence and risk factors for mortality of vertebral osteomyelitis: a retrospective analysis using the Japanese diagnosis procedure combination database. *BMJ Open* **3**, e002412 10.1136/BMJOPEN-2012-002412 (2013).
22. Veljanoski, D. et al. Spinal infections in the north-east of Scotland: a retrospective analysis. *Ann. R. Coll. Surg. Engl.* **105**, 428–433 10.1308/RCSANN.2022.0062 (2023).
23. Sircar, K. et al. Risk factors for neurologic deficits in patients with spinal epidural abscess: an analysis of one-hundred-forty cases. *Glob Spine J.* 10.1177/21925682231194467 (2023).
24. Ross, J. S. et al. Association between peridural scar and recurrent radicular pain after lumbar discectomy: magnetic resonance evaluation. *Neurosurgery*. **38**, 855–863 (1996).
25. Sircar, K. et al. evaluation of classification systems and their correlation with clinical and quality-of-life parameters in patients with surgically treated spondylo. (2023).

As a result, the References have been renumbered.

The original Article has been corrected.

