# Supplementary material for: A post-hoc analysis of risk factors for poor quality of life after surgical treatment of spondylodiscitis
Source: Sci Rep. 2024 Nov 17;14:28365. doi: 10.1038/s41598-024-79828-8 (PMC11570602; doi:10.1038/s41598-024-79828-8)
Supplement: Supplementary file 1 — Supplementary Material 1 [file 41598_2024_79828_MOESM1_ESM.docx]

**Supplemental Table 2:** Details of the 22 patients with a malignant disease. Active malignant disease was defined by diagnosis, treatment, recurrence, or progress within the preceding two years before diagnosis of spondylodiscitis, or a metastasized malignant disease.

| **Factor** | **Group 1 (ODI < 35)**  **n=10** | **Group 2 (ODI ≥ 35)**  **n= 12** | **P-Value** |
| --- | --- | --- | --- |
|  |  |  |  |
| Status of neoplasm activity |  |  |  |
| active | 6 | 8 | 0.34 |
| inactive | 4 | 4 |  |
|  |  |  |  |
|  |  |  |  |
| Neoplasm entity |  |  |  |
| solid | 8 | 9 | 0.47 |
| hematologic | 2 | 2 |  |
| both | 0 | 1 |  |
