# Supplementary material for: A post-hoc analysis of risk factors for poor quality of life after surgical treatment of spondylodiscitis
Source: Sci Rep. 2024 Nov 17;14:28365. doi: 10.1038/s41598-024-79828-8 (PMC11570602; doi:10.1038/s41598-024-79828-8)
Supplement: Supplementary file 2 — Supplementary Material 2 [file 41598_2024_79828_MOESM2_ESM.docx]

**Supplemental Table 1:** The Oswestry Disability Index. Each of the ten items has a value of zero to five. The sum of all ten items is multiplied by two. Zero points indicate no functional impairment, whereas the maximum of 100 points indicates complete functional impairment.

|  |  |
| --- | --- |
| **ITEM** | **POINTS** |
| 1. PAIN INTENSITY |  |
| I can tolerate the pain I have without having to use pain killers | 0 |
| The pain is bad but I manage without taking pain killers | 1 |
| Pain killers give complete relief from pain | 2 |
| Pain killers give moderate relief from pain | 3 |
| Pain killers give very little relief from pain | 4 |
| Pain killers have no effect on the pain and I do not use them | 5 |
| 2. PERSONAL CARE (e.g. Washing, Dressing) |  |
| I can look after myself normally without causing extra pain | 0 |
| I can look after myself normally but it causes extra pain | 1 |
| It is painful to look after myself and I am slow and careful | 2 |
| I need some help but manage most of my personal care | 3 |
| I need help every day in most aspects of self-care | 4 |
| I don’t get dressed, I was with difficulty and stay in bed | 5 |
| 3. LIFTING |  |
| I can lift heavy weights without extra pain | 0 |
| I can lift heavy weights but it gives extra pain | 1 |
| Pain prevents me from lifting heavy weights off the floor, but I can manage if they are conveniently positioned, i.e. on a table | 2 |
| Pain prevents me from lifting heavy weights, but I can manage light to medium weights if they are conveniently positioned | 3 |
| I can lift very light weights | 4 |
| I cannot lift or carry anything at all | 5 |
| 4. WALKING |  |
| Pain does not prevent me walking any distance | 0 |
| Pain prevents me walking more than one mile | 1 |
| Pain prevents me walking more than ½ mile | 2 |
| Pain prevents me walking more than ¼ mile | 3 |
| I can only walk using a stick or crutches | 4 |
| I am in bed most of the time and have to crawl to the toilet | 5 |
| 5. SITTING |  |
| I can sit in any chair as long as I like | 0 |
| I can only sit in my favorite chair as long as I like | 1 |
| Pain prevents me from sitting more than one hour | 2 |
| Pain prevents me from sitting more than ½ hour | 3 |
| Pain prevents me from sitting more than 10 minutes | 4 |
| Pain prevents me from sitting at all | 5 |
| 6. STANDING |  |
| I can stand as long as I want without extra pain | 0 |
| I can stand as long as I want but it gives me extra pain | 1 |
| Pain prevents me from standing for more than one hour | 2 |
| Pain prevents me from standing for more than 30 minutes | 3 |
| Pain prevents me from standing for more than 10 minutes | 4 |
| Pain prevents me from standing at all | 5 |
| 7. SLEEPING |  |
| Pain does not prevent me from sleeping well | 0 |
| I can sleep well only by using medication | 1 |
| Even when I take medication, I have less than 6 hrs sleep | 2 |
| Even when I take medication, I have less than 4 hrs sleep | 3 |
| Even when I take medication, I have less than 2 hrs sleep | 4 |
| Pain prevents me from sleeping at all | 5 |
| 8. SOCIAL LIFE |  |
| My social life is normal and gives me no extra pain | 0 |
| My social life is normal but increases the degree of pain | 1 |
| Pain has no significant effect on my social life apart from limiting my more energetic interests | 2 |
| Pain has restricted my social life and I do not go out as often | 3 |
| Pain has restricted my social life to my home | 4 |
| I have no social life because of pain | 5 |
| 9. TRAVELLING |  |
| I can travel anywhere without extra pain | 0 |
| I can travel anywhere but it gives me extra pain | 1 |
| Pain is bad, but I manage journeys over 2 hours | 2 |
| Pain restricts me to journeys of less than 1 hour | 3 |
| Pain restricts me to short necessary journeys under 30 minutes | 4 |
| Pain prevents me from traveling except to the doctor or hospital | 5 |
| 10. EMPLOYMENT/ HOMEMAKING |  |
| My normal homemaking/ job activities do not cause pain. | 0 |
| My normal homemaking/ job activities increase my pain, but I can still perform all that is required of me. | 1 |
| I can perform most of my homemaking/ job duties, but pain prevents me from performing more physically stressful activities (e.g. lifting, vacuuming). | 2 |
| Pain prevents me from doing anything but light duties. | 3 |
| Pain prevents me from doing even light duties. | 4 |
| Pain prevents me from performing any job or homemaking chores. | 5 |
